# Spotted Fever Group Rickettsiae in Ticks, Morocco 

**DOI:** 10.3201/eid1407.070096

**Published:** 2008-07

**Authors:** Mhammed Sarih, Cristina Socolovschi, Najma Boudebouch, Mohammed Hassar, Didier Raoult, Philippe Parola

**Affiliations:** * Institut Pasteur du Maroc, Casablanca, Morocco; † World Health Organization Collaborative Center for Rickettsioses and Other Arthropod Borne Bacterial Diseases, Marseille, France; 1 These authors contributed equally to this article.

**Keywords:** Ticks, Morocco, rickettsia, spotted fever, research

## Abstract

A total of 370 ticks, encompassing 7 species from 4 genera, were collected during 2002–2006 from domestic animals and vegetation in the Taza region of northeastern Morocco. Rickettsial DNA was identified in 101 ticks (27%) by sequencing PCR products of fragments of the citrate synthase and outer membrane protein genes of *Rickettsia* spp. Seven rickettsiae of the spotted fever group were identified, including 4 pathogens: *R*. *aeschlimannii* in *Hyalomma marginatum marginatum*, *R. massiliae* in *Rhipicephalus sanguineus*, *R. slovaca* in *Dermacentor marginatus*, and *R. monacensis* in *Ixodes ricinus.* Two suspected pathogens were also detected (*R. raoultii* in *D. marginatus* and *R. helvetica* in *I. ricinus)*. An incompletely described *Rickettsia* sp. was detected in *Haemaphysalis* spp. ticks.

## Abstract 2

Identified rickettsiae were 4 pathogens, 2 suspected pathogens, and 1 incompletely described species.

Tick-borne rickettsioses are infections caused by obligate intracellular gram-negative bacteria of the spotted fever group (SFG) in the genus *Rickettsia* and the order Rickettsiales. These zoonoses are now recognized as emerging vector-borne infections worldwide (*1*,*2*). They share characteristic clinical features, including fever, headache, rash, and occasional eschar formation at the site of the tick bite. Although these diseases have been known for a long time, they have been poorly investigated in northern Africa, including Morocco (*2*).

Two human tick-borne SFG rickettsioses are known to occur in Morocco. Mediterranean spotted fever, caused by *Rickettsia conorii conorii*, is transmitted by the brown dog tick, *Rhipicephalus sanguineus*, which is well adapted to urban environments and is endemic to the Mediterranean area (*2*). In Morocco, clinicians usually consider patients with spotted fever as having Mediterranean spotted fever. However, in 1997, Beati et al. isolated a new rickettsia, *R*. *aeschlimannii,* from *Hyalomma marginatum marginatum* ticks collected in Morocco (*3*). In 2002, human infection with this rickettsia was reported in a patient returning from Morocco to France (*4*).

To date, all studies on rickettsioses conducted in Morocco have been based on only clinical and serologic features. However, the number of representatives of the genus *Rickettsia* and the number of newly described rickettsioses have increased in recent decades because of improved cell culture isolation techniques and extensive use of bacterial detection and identification by molecular biologic techniques (*2*). Comparison of the sequences of PCR-amplified fragments of genes encoding 16S rRNA, citrate synthase (*glt*A), or outer membrane protein (*ompA*) has become a reliable method for identifying rickettsiae in arthropods, including ticks (*1*). Therefore, our aim was to detect and characterize rickettsiae in hard ticks collected in Morocco by using PCR and sequence analysis of amplified products and to discuss their potential threat for humans and animals.

## Materials and Methods

### Collection and Identification of Ticks

From April 2002 through March 2006, ticks were collected from domestic animals (livestock and dogs) and by flagging vegetation at sites in the Taza region in northeastern Morocco. These sites were located between the towns of Babboudir and Babezhare, (34°12′48.81′′N, 4°0′55.63′′W) in the Atlas Mountains, situated 40 km from the city of Taza and 90 km from the city of Fez. All ticks collected were adults and morphologically identified to the species or genus level by using standard taxonomic keys. Ticks were kept in ethanol at room temperature until DNA was extracted in the Laboratoire des Maladies Vectorielles, Institut Pasteur du Maroc, Casablanca, Morocco. DNA samples were thereafter sent to the Unité des Rickettsies in Marseille, France.

### PCR Detection and Identification of *Rickettsia* spp.

Ticks were rinsed with distilled water for 10 min, dried on sterile filter paper in a laminar flow hood, and crushed individually in sterile Eppendorf (Hamburg, Germany) tubes. DNA was extracted by using the QIAamp Tissue Kit (QIAGEN, Hilden, Germany) according to the manufacturer’s instructions. Rickettsial DNA was detected by PCR by using primers *Rp* CS.409p and *Rp* CS.1258n (Eurogentec, Seraing, Belgium), which amplify a 750-bp fragment of the *gltA* gene of *Rickettsia* spp. as described (*5*). All ticks positive for *gltA* were tested for the *ompA* gene of *Rickettsia* spp. by using primers Rr. 190.70 and Rr. 190.701, which amplify a 629–632-bp fragment (*5*). A negative control (distilled water instead of tick DNA template) and a positive control (DNA from *R*. *montanensis*) were included in each test. All PCRs were conducted in Marseille by using the GeneAmp PCR System 2400 and 9700 thermal cyclers (PerkinElmer, Waltham, MA, USA). Amplification products were analyzed after electrophoresis on a 1% agarose gel stained with ethidium bromide. To identify detected *Rickettsia* spp., PCR products were purified and sequencing was performed as described (*5*). All sequences obtained were assembled and edited with Auto Assembler software version 1.4 (PerkinElmer). Sequences were analyzed by BLAST (www.ncbi.nlm.nih.gov/blast/Blast.cgi) sequencing analysis of sequences in the GenBank database.

### Molecular Identification of Ticks

To help identify the ticks at the species level, molecular tools were used for some ticks that had not been morphologically identified at the species level and that were positive for rickettsiae. Amplification by PCR with T1B and T2A primers and sequencing of a 338-bp amplified fragment of the 12S rRNA gene of the ticks were performed as described (*6*).

## Results

A total of 370 specimens representing 7 species and 4 genera of ticks were collected. Tick species identified by taxonomic keys included *Rh*. *sanguineus* (106 specimens), *Rh*. *bursa* (76), *Rh*. *turanicus* (25), *Haemaphysalis sulcata* (79), *Ha*. *punctata* (6), *Ixodes ricinus* (14), and *Dermacentor marginatus* (11) (Figure). Some ticks, including engorged females or damaged specimens, were identified to genus only (18 *Haemaphysalis* sp. and 35 *Hyalomma* sp.). Most ticks (337) were collected from domestic animals; the rest were collected by flagging of vegetation (Table).

**Figure:**
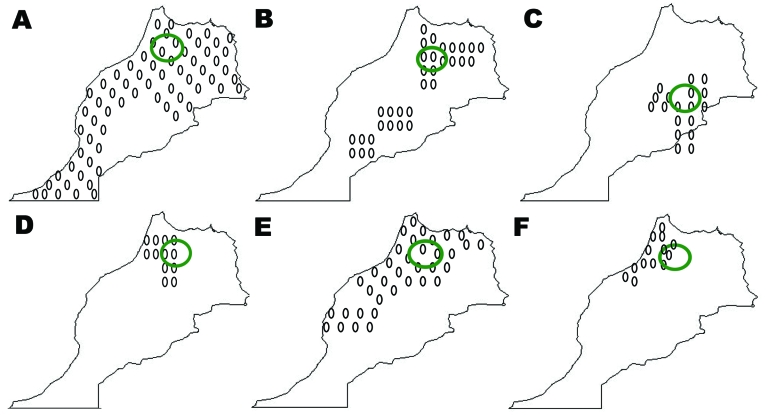
Distribution of ticks in Morocco from which rickettsial DNA was detected by PCR. A) *Rhipicephalus sanguineus*, B) *Haemaphysalis sulcata*, C) *Ha*. *punctata*, D) *Ixodes ricinus*, E) *Hyalomma marginatum marginatum*, F) *Dermacentor marginatus.* Green circles indicate areas where ticks were collected and found to harbor rickettsiae. Ovals indicate distribution of each tick species in which rickettsial DNA was detected by PCR.

**Table:** Detection and identification of spotted fever group *Rickettsia* spp. from ticks collected in Morocco, by PCR and DNA sequencing

Tick species (no. specimens tested)	Host	Rickettsial gene targeted/no. ticks positive by PCR/no. examined*	Identification by gene sequence (no. identified/no. tested)	GenBank accession no.
*Dermacentor marginatus* (11)	Vegetation	*glt*A/6/11	*R. slovaca* (5/6), *R. raoultii* (1/6)	U59725, DQ365803
		*omp*A/6/6	*R. slovaca* (5/6), *R. raoultii* (1/6)	U43808, DQ365799
*Hyalomma marginatum marginatum* (35)	Domestic animals	*glt*A/3/35	*R. aeschlimannii*	U59722
		*omp*A/3/3	*R. aeschlimannii*	DQ379982
*Haemaphysalis sulctata* (79)	Domestic animals	*glt*A/61/79	*“Rickettsia endosymbiont of Ha. Sulctata” (“R. kastelanii”)*	DQ081187
		*omp*A/0/61	–	–
*Ha. punctata* (6)	Domestic animals	*glt*A/3/6	*“Rickettsia endosymbiont of Ha. Sulctata” (“R. kastelanii”)*	DQ081187
		*omp*A/0/3		
*Haemaphysalis* sp. (18)	Vegetation	*glt*A/14/18	*“Rickettsia endosymbiont of Ha. Sulctata” (“R. kastelanii”)*	DQ081187
		*omp*A/0/14	–	–
*Ixodes ricinus* (14)	Vegetation	*glt*A/9/14	*R. monacensis* (5/9), *R. helvetica* (4/9)	AF140706, U59723
		*omp*A/5/9	*R. monacensis* (5/9)	AJ427885
*Rhipicephalus sanguineus* (106)	Domestic animals	*glt*A/5/106	*R. massiliae*	U59720
		*omp*A/5/5	*R. massiliae*	U43792
*Rh. bursa* (76)	Domestic animals	*glt*A/0/76	–	–
*Rh. turanicus* (25)	Domestic animals	*glt*A/0/25	–	–

*gltA, citrate synthase A; *omp*A, outer membrane protein A. Only ticks positive for the *glt*A gene were tested for the *omp*A gene.

Rickettsial DNA was detected in 101 (27%) of 370 ticks by using a *gltA* PCR. Three (8.6%) of 35 *Hyalomma* spp. ticks contained rickettsia DNA with a *glt*A gene fragment that was 99.1% (765/772 bp) similar to that of *R*. *aeschlimannii* and 100% similar to the *omp*A gene of *R*. *aeschlimannii*. A 237-bp fragment of tick mitochondrial 12S rDNA gene was obtained from one of the *R*. *aeschlimannii*–infected ticks. The sequence of this fragment enabled definitive identification of the tick to the species level, with 100% similarity to *H*. *marginatum marginatum* (GenBank accession no. AF150034).

Five (4.7%) of 106 *Rh*. *sanguineus* ticks were positive for rickettsial DNA by PCR. For all samples, sequence analyses showed 99.8% (636/637) similarity with the *glt*A sequence and 100% similarity with the *omp*A sequence of *R*. *massiliae*. One *R*. *massiliae*–infected tick was evaluated by PCR amplification of the tick mitochondrial 12S rDNA gene; sequence analyses showed 99.6% (235/236) similarity to the corresponding 12S rDNA of *Rh*. *sanguineus* (GenBank accession no. AF133056).

A total of 5 (45.5%) of 11 *D*. *marginatus* ticks contained a rickettsia with a nucleotide sequence of *glt*A that was 99.2% (635/640 bp) similar to *R*. *slovaca* and 100% (533/533 bp) similar to the *omp*A sequence of *R*. *slovaca*. Rickettsial DNA was detected in 1 other specimen of *D*. *marginatus*. Amplified *glt*A and *omp*A fragments were sequenced and showed 99.3% (560/564 bp) similarity with the *glt*A gene of *R*. *raoultii* and 100% similarity with the *omp*A gene of *R*. *raoultii*.

Five (35.7%) of 14 specimens of *I*. *ricinus* were positive by *glt*A PCR. Sequence analyses showed 100% homology with the corresponding *glt*A sequence of *R*. *monacensis*. *Omp*A sequences were obtained and showed 99.7% (585/587 bp) similarity with the corresponding sequence of *R*. *monacensis*. Four (28.6%) of 14 *I*. *ricinus* ticks contained rickettsia with nucleotide sequences of *glt*A with 99.8% (633/634 bp) similarity to *R*. *helvetica*. The primer set Rr.190.70p-Rr.190.701n failed to amplify an *omp*A product in any specimens that were positive for the *glt*A gene of *R*. *helvetica.*

Sixty-one (77.2%) of 79 *Ha*. *sulcata* ticks, 3 (50%) of 6 *Ha*. *punctata* ticks, and 14 (77.7%) of 18 *Haemaphysalis* spp. ticks were positive by PCR for the primer set *Rp* CS.409p and *Rp* CS.1258n for the *glt*A gene. The *glt*A sequences obtained were different from all known *Rickettsia* spp. sequences deposited in GenBank. The most closely related sequence of *glt*A was designated “Ricketttsia endosymbiont of *Haemaphysalis sulctata*” (99.4% similarity; 484/487 bp). The next most closely related sequence of *glt*A, with 96% similarity, was *R*. *felis*. Results of the PCR with the *omp*A primer set Rr.190.70p-Rr.190.701n were negative for all *Haemaphysalis* spp. ticks that were positive for the *glt*A gene.

None of the *Rh*. *bursa* and *Rh*. *turanicus* ticks harbored rickettsiae. All GenBank accession numbers used to compare sequences obtained from ticks are shown in the Table.

## Discussion

Before this study, only 2 SFG rickettsiae pathogenic to humans had been described in Morocco, *R*. *conorii conorii*, the agent of Mediterranean spotted fever, and the recently described *R*. *aeschlimannii* (*2*,*3*). In our study, in addition to *R*. *aeschlimannii,* we identified 3 other SFG pathogenic rickettsiae in Morocco: *R*. *massiliae*, *R*. *slovaca*, and *R*. *monacensis*. Furthermore, 2 tick-borne SFG *Rickettsia* spp. presumptively associated with human illnesses, *R*. *helvetica* and *R*. *raoultii*, and an undescribed bacterium have been identified.

DNA extraction and PCR were performed in different locations (Morocco and France), and all results were supported by 2 sets of primers. The *gltA* primers used in the first screening are known to amplify all known tick-borne rickettsiae (*7*). A second set of primers targeting the *ompA* gene was used to confirm positive results, although some rickettsia (e.g., *R*. *helvetica*) cannot be amplified by using this set. There were no cases in which multiple species of rickettsiae were detected in an infected tick, as in most of the similar molecular surveys published (*1*,*2*). Our results did not address prevalence and distribution of rickettsiae detected. Systematic sampling was not conducted. Also, some tick samples tested with rickettsial primers have not been tested with tick primers in parallel. Therefore, inhibitors that could be responsible for false-negative results and underestimation of infection rates cannot be ruled out.

*R*. *aeschlimannii* was isolated from *H*. *marginatum* *marginatum* ticks collected in Morocco in 1997 (*3*). This rickettsia has also been detected in *H*. *marginatum rufipes* ticks in Zimbabwe, Niger, and Mali; in *H*. *marginatum marginatum* in Portugal, Croatia, Spain, Greece, Algeria, and Egypt; and in both ticks in Corsica (*2*,*8*,*9*). *H*. *marginatum marginatum* is also known as the Mediterranean *Hyalomma* and may represent up to 42% of ticks found on cattle in Morocco. This tick is also a suspected reservoir of *R*. *aeschlimannii* because transstadial and transovarial transmission have been reported (*8*). As a result, the distribution of *R*. *aeschlimannii* may parallel that of *H*. *marginatum marginatum*.

In 2002, the pathogenic role of infection with *R*. *aeschlimannii* was demonstrated by PCR and serologic testing in a patient who returned to France from Morocco (*4*). Clinical signs in this 36-year-old man were fever, generalized maculopapular rashes, and a vesicular lesion of the ankle that became necrotic and resembled the typical tache noire of Mediterranean spotted fever. A second case was identified in a patient in South Africa in 2002 (*10*). This patient had an eschar around the attachment site. No additional symptoms developed, and treatment with antimicrobial drugs may have prevented progression of the syndrome.

A total of 4.7% of the *Rh*. *sanguineus* ticks tested were infected by *R*. *massiliae*. This rickettsia was isolated from *Rh*. *sanguineus* ticks collected near Marseille, France, in 1992 (*11*). It has been also found in *Rh*. *sanguineus* and *Rh*. *turanicus* in Greece, Spain, Portugal, Switzerland, central Africa, and Mali (*2*,*12*,*13*). Eremeeva et al. (*14*) recently reported detection and isolation of *R*. *massiliae* from 2 of 20 *Rh*. *sanguineus* ticks collected in eastern Arizona in the United States. *R*. *massiliae* may be commonly associated with these ticks, which are distributed worldwide. Transstadial and transovarial transmission of rickettsia in ticks has been reported (*13*).

In 2003, serologic findings from Spain showed that in 5 of 8 serum samples titers against *R*. *massiliae* were higher than those against *R*. *conorii*, the agent of Mediterranean spotted fever (*12*). The authors analyzed clinical symptoms of patients with strong serologic reactions against *R*. *massiliae* antigens but did not find relevant clinical differences between these patients and those with Mediterranean spotted fever. However, it is generally recognized that there are relatively few clinical differences among the different spotted fever diseases, and these differences are occasionally not taken into account by clinicians when reporting clinical data of patients (*12*). The only confirmed case of a person infected with *R*. *massiliae* was a patient hospitalized in Sicily, Italy. This patient had fever, a maculopapular rash on the palms of his hands and the soles of his feet, an eschar, and hepatomegaly. The strain of *R*. *massiliae* was isolated in Vero cells in 1985 and stored for 20 years in Sicily, but was not definitively identified until 2005 at the Unité de Rickettsies in Marseille, France (*15*).

The third SFG pathogenic rickettsia found in our study was *R*. *slovaca* in 5 (45.5%) of 11 *D*. *marginatus*. *R*. *slovaca,* which was identified in *Dermacentor* spp. ticks in Slovakia in 1968, has been subsequently found in *D*. *marginatus* and *D*. *reticulatus* in France, Switzerland, Portugal, Spain, Armenia, Poland, Bulgaria, Croatia, Russia, and Germany (*2*,*16*). These ticks may act as vectors and reservoirs of *R*. *slovaca,* which is maintained in ticks through transstadial and transovarial transmission (*17*). Human infection with *R*. *slovaca* was reported in France in 1997. Patients with similar clinical signs were observed in Spain, Bulgaria, and Hungary, where the syndrome was known as tick-borne lymphadenopathy or *Dermacentor*-borne necrosis erythema lymphadenopathy because of eschar at the tick bite site in the scalp and cervical lymphadenopathy (*2*,*18*–*20*). The incubation period ranges from 4 to 15 days. Low-grade fever and rash were present. The acute disease can be followed by fatigue and residual alopecia at the bite site (*16*,*21*). Recently, Gouriet et al. reported 14 new cases with tick-borne lymphadenopathy and *Dermacentor*-borne necrosis erythema lymphadenopathy in southern France during January 2004–May 2005 (*22*). In this group, tick-borne lymphadenopathy occurred mainly in young children and women and during the colder months (*22*). Overall, data in our study indicate that clinicians should be aware that this tick-related disorder may be found in Morocco.

*R*. *raoultii* is a recently described SFG rickettsia (*23*). In 1999, three new rickettsial genotypes, RpA4, DnS14, and DnS28, were identified in ticks collected in Russia by using PCR amplification and sequencing of 16S rDNA, *gltA*, and *ompA* genes. Genotypes identical to DnS14, DnS28, and RpA4 were thereafter detected in various areas in Russia and Kazakhstan in *D*. *reticulatus*, *D*. *marginatus*, and *D*. *silvarum* (*24*), in Germany and Poland in *D*. *reticulatus* (*25*,*26*), and in Spain, France, and Croatia in *D*. *marginatus* (*23*). Recently, cultivation of 2 rickettsial isolates genetically identical to *Rickettsia* sp. genotype DnS14, two rickettsial isolates genetically identical to *Rickettsia* sp. genotype RpA4, and 1 rickettsial isolate genetically identical to *Rickettsia* sp. genotype DnS28 was described (*23*). These isolates have been shown to fulfill the requirements for their classification within a new species, *R*. *raoultii*, by using multigene sequencing (16S rDNA, *gltA*, *ompA*, *ompB*, *sca4*, *ftsY*, and *rpoB* genes) and serotyping techniques (*23*,*27*). In our study, we detected *R*. *raoultii* in *D*. *marginatus* in Morocco. This tick is found in the cooler and more humid areas of the Mediterranean region associated with the Atlas Mountains. It is restricted to small areas of Morocco and Tunisia (*28*). Detection of *R*. *raoultii* in Morocco is of clinical relevance because it is suspected to be a human pathogen. In 2002, it was detected in *D*. *marginatus* obtained from a patient in France in whom typical clinical symptoms of tick-borne lymphadenopathy developed (*23*).

*R*. *helvetica* is another species identified in Morocco in this study. It is one of the few SFG species in which a commonly used o*mp*A primer set does not amplify a PCR product (*7*,*29*). However, sequencing *glt*A enabled definitive identification. *R*. *helvetica* was isolated in Switzerland from *I*. *ricinus* in 1979 and has been identified in many European countries, where the tick is both a vector and a reservoir (*2*). The distribution of *R*. *helvetica* is not limited to Europe but extends into Asia (*30*). Our data show that the distribution of this bacterium extends into northern Africa. A small population of *I*. *ricinus* is present in Tunisia, Algeria, and Morocco. Our study was conducted in Taza, a humid area in the middle of the Atlas Mountains, which was the only site in Morocco that contained *I*. *ricinus* ticks (*2*).

*R*. *helvetica* was considered to be a nonpathogenic rickettsia for ≈20 years after its discovery. However, in 1999 it was implicated in fatal perimyocarditis in patients in Sweden (*31*). The authors of this study subsequently reported a controversial association between *R*. *helvetica* and sarcoidosis in Sweden (*32*) and found *R*. *helvetica* DNA in human aortic valves (*33*). However, the validity of these associations has been questioned by some rickettsiologists (*2*), and additional studies did not detect antibodies to rickettsia in a group of Scandinavian sarcoidosis patients (*34*). In 2000, seroconversion for *R*. *helvetica* was described in a patient in France with a nonspecific febrile illness (*35*). Serologic data, including cross-absorption and Western blotting, supported *R*. *helvetica* as the cause of disease. During 2003–2007, serologic findings in tickbite patients or in patients with fever of unknown origin from Switzerland, Italy, France, and Thailand were suggestive of acute or past *R*. *helvetica* infection (*5*,*36*). The few patients with a serology-based diagnosis had relatively mild, self-limited illnesses associated with headache and myalgias, and had a rash or eschar less frequently. Additional evaluation and isolation of the bacterium from clinical samples are needed to confirm the pathogenicity of *R*. *helvetica*.

We have detected in *I*. *ricinus* ticks a bacterium known as *R*. *monacensis* that was isolated from *I*. *ricinus* collected in 1998 in a park in Munich, Germany (*37*). This rickettsia is also found in the literature by other names such as the Cadiz agent found in Spain and *Rickettsia* IRS3 and IRS4, detected in Slovakia and Bulgaria. More recently, it has been identified in *I*. *ricinus* in Hungary (*38*). Recently, 2 human cases of infection with *R*. *monacensis* were documented in Spain (*39*). Investigators isolated this agent from the blood of 2 patients with Mediterranean spotted fever–like illnesses. The first patient was an 84-year-old man from La Rioja, Spain. He had fever and maculopapular rash without any inoculation eschar. The second patient was a 59-year-old woman from the Basque region of Spain. She had a history of a tickbite, fever, and a rash at the tickbite site (*39*). With our results, *R*. *monacensis* joins the list of autochthonous *Rickettsia* spp. confirmed as human pathogens in Morocco.

A total of 69% of *Haemaphysalis* spp. ticks tested harbored an incompletely described rickettsia. A closely related *glt*A sequence was found in GenBank as *Rickettsia endosymbiont of Haemaphysalis sulctata*. Duh et al. detected this bacterium in *Ha. sulcata* ticks collected from sheep and goats in southern Croatia (*40*). Using molecular analysis of the complete *glt*A gene and a portion of *omp*B, these authors detected this bacterium in 795 (22.8%) ticks tested. Similar to our findings, these researchers could not amplify DNA by PCR for the *omp*A gene with the primers Rr. 190.70-Rr. 190.701. Identification and isolation of this bacterium are needed until the name provisionally proposed by Duh et al, “*R*. *kastelanii*” (*40*), is accepted (*41*).

These findings demonstrate that species of ticks and several pathogens causing tick-transmitted diseases may be prevalent in the same area. Our study also detected *R*. *slovaca*, *R*. *helvetica*, *R*. *monacensis*, *R*. *raoultii*, and an incompletely described rickettsia in Morocco. Clinicians in Morocco and those who may see patients returning from this country should be aware that many species of rickettsiae are present in this region and should consider a range of spotted fever rickettsial diseases in differential diagnosis of patients with febrile illnesses. Our data increase information on distribution of SFG rickettsiae in Morocco. Additional studies are needed to determine the epidemiologic and clinical relevance of different rickettsioses in this region.
